# Respiratory Rate Estimation by Using ECG, Impedance, and Motion Sensing in Smart Clothing

**DOI:** 10.1007/s40846-017-0247-z

**Published:** 2017-07-01

**Authors:** Chien-Lung Shen, Tzu-Hao Huang, Po-Chun Hsu, Ya-Chi Ko, Fen-Ling Chen, Wei-Chun Wang, Tsair Kao, Chia-Tai Chan

**Affiliations:** 1 Department of Biomedical Engineering, National Yang-Ming University, No.155, Sec.2, Linong Street, Taipei, 112 Taiwan, ROC; 2 Taiwan Textile Research Institute, No.6, Chengtian Rd., Tucheng Dist., New Taipei City, 23674 Taiwan, ROC

**Keywords:** Textile electrode, Multiple sensors, Smart clothing, Respiration rate

## Abstract

The needs for light-weight and soft smart clothing in homecare have been rising since the past decade. Many smart textile sensors have been developed and applied to automatic physiological and user-centered environmental status recognition. In the present study, we propose wearable multi-sensor smart clothing for homecare monitoring based on an economic fabric electrode with high elasticity and low resistance. The wearable smart clothing integrated with heterogeneous sensors is capable to measure multiple human biosignals (ECG and respiration), acceleration, and gyro information. Five independent respiratory signals (electric impedance plethysmography, respiratory induced frequency variation, respiratory induced amplitude variation, respiratory induced intensity variation, and respiratory induced movement variation) are obtained. The smart clothing can provide accurate respiratory rate estimation by using three different techniques (Naïve Bayes inference, static Kalman filter, and dynamic Kalman filter). During the static sitting experiments, respiratory induced frequency variation has the best performance; whereas during the running experiments, respiratory induced amplitude variation has the best performance. The Naïve Bayes inference and dynamic Kalman filter have shown good results. The novel smart clothing is soft, elastic, and washable and it is suitable for long-term monitoring in homecare medical service and healthcare industry.

## Introduction

Over the last decade, the call for light-weight, soft, wearable devices have gradually increased. Many smart textile sensors have been developed and applied to automatic physiological and user-centered environmental status recognition: for example, T-shirt with sensors integrated for wearable cardiopulmonary monitoring [1], wearable electrocardiogram (ECG) Recorder with acceleration sensors [2], real-time cardiac monitoring on smartphone [3], the conductive yarn for ECG sensing [4–6], the piezoresistive yarn for motion sensing [4, 5], the flexible circuit board for the connection between chip and sensor network in clothing [4, 5], the wearable antenna for wireless data transmission [4], the magnetic inductive textile coil for standing, sitting, slow walking fast walking, and jogging detection [7], and the bendable and wearable textile sensors of magnetic induction for cardiorespiratory monitoring [8, 9]. Most of these sensors are designed to detect only a specific signal. However, for many applications, multiple sensors measurement and comprehensive sensory integration are needed. Scalisi et al. used inkjet printed flexible electrodes to measure multiple surface electromyography (sEMG) signals [10]. Or in homecare field, fallen alert, health monitoring, and exercise management require not only the measurement of movement, but also that of ECG, heart rate, temperature, and respiration. Therefore, clothing with integrated sensory network becomes important. In this work, we approach this problem by studying the estimation of respiratory rate. Because the respiration signal manifests in miscellaneous wearables sensors, it is an illustrative scenario to demonstrate how smart clothing can take advantage of these diverse sensors for better homecare and health monitoring.

Many respiration detecting methods have been investigated for smart clothing and bands, such as magnetic induction monitoring [7–9, 11], photoplethysmogram (PPG) [11–14], peripheral arterial tonometry (PAT) [15, 16], electric impedance plethysmography (EIP) [15, 17–20], respiratory inductive plethysmography (RIP) [20–22], piezoresistive sensors [23], accelerometer [22, 24–30], and ECG derived respiration (EDR) [15, 22, 30]. However, estimating respiratory rate remains difficult. Because of the non-stationary nature of respiration signals and the overlapped frequency band of the movement artifact, baseline drifting, ECG, and respiratory signal, each of the sensing methods described above has its own preferable working domain. Respiratory rate estimations using PPG, ECG, and EIP work only in static situation, because movements during dynamic activities induce slipping between electrodes and device and therefore influence the signal quality. Though the usage of accelerometer allows minor movement artifacts during measurement, it is still hard to extract small respirations signal from large movements. On the other hand, both RIP and piezoresistive sensors suffer from large baseline drifting, if the sensor does not contact the skin well; even though tight wearable bands tend to perform well, the users would more easily feel uncomfortable.

Algorithms have been developed to merge multiple sensors for more accurate, and stable respiration estimation, such as respiratory rate and heart rate estimation by the adaptive Kalman filter from three magnetic induction sensors [31], the data fusion to enhance bio-signal by bendable noncontact magnetic inductive sensors and PPG [11], the data fusion for EDR and PAT [15], the probabilistic inference using baseline drifting, the amplitude modulation and frequency modulation of PPG [12, 32], and the sensor fusion of accelerometer and gyro-sensor [25]. In [11], they pointed out that the fusion of bendable noncontact magnetic inductive sensors and PPG at the same measurement location could allow the possibility of motion artifact cancellation. In [31], the worst case obtained mean errors of −0.2 rcpm by using adaptive Kalman filter and three magnetic induction sensors and they point out that adaptive Kalman filter can continuously improve the ability to separate the desired signals from the raw sensor data. In [15], the amplitude modulation, frequency modulation, and pulse amplitude of PAT are extracted and combined by Kalman filter, in which the measurement noise covariance is updated according to signal quality metrics. They achieve average root-mean-squared (RMS) error over 30 subjects of 2.7 respiratory cycles per minute (rcpm). In [32], respiratory induced frequency variation (RIFV), respiratory induced amplitude variation (RIAV), and respiratory induced intensity variation (RIIV) are extracted from PPG sensor and the respiratory rate is calculated as their mean value after the removal of outliers. The result has average RMS error of 3 rcpm. In [12], the baseline drifting, amplitude modulation, and frequency modulation of PPG are extracted and Gaussian process regression is used to offline infer a probabilistic model for respiratory rate estimation. The data fusion is achieved by interpolating the mean estimates according to the prediction variance of the three Gaussian process regression models. The average of mean respiratory absolute error in [12] is about 2.7 rcpm. In [25], accelerometer and gyro sensor are used to extract the Euler angles of respiratory movement. Then, with the Euler angles expressed in quaternions, a Kalman filter is used to fuse the quaternions from acceleration and angular velocity. Finally, the merged quaternions are transferred back to the Euler angles, which are considered as the respiratory signal. The error rates they achieve are 4.6 and 9.54% in the treadmill and leg press exercises, respectively. As shown in Table 1, the fusion of respiration data can lead to more accurate and stable respiratory rate estimation.

**Table 1:** The comparison of different respiratory estimations

References	Merge methods	Signals	Results
[32]	Adaptive Kalman Filter	Three magnetic induction sensors	Mean error: −0.2 rcpm
[15]	Kalman filter	AM, FM, and pulse amplitude of PAT	RMS error: 2.7 rcpm
[33]	Average	RIFV, RIAV, RIIV of PPG	RMS error: 3 rcpm
[12]	Gaussian process regression	Baseline, AM, FM of PPG	Absolute error: 2.7 rcpm
[26]	Kalman filter	Accelerometer and gyro	Error rate in treadmill task: 4.6% In leg press task: 9.54%

*AM* amplitude modulation; *FM* frequency modulation; *PAT* peripheral arterial tonometry; *rcpm* respiratory cycles per minute; *RIFV* respiratory induced frequency variation; *RIAV* respiratory induced amplitude variation; *RIIV* respiratory induced intensity variation; *PPG* photoplethysmogram; *RMS* root mean square

In the present study, we propose multi-sensor smart clothing for homecare monitoring based on our novel *CB/PBT/PET*-*wrapped* 1 *AGposs yarn,* 2 an economic fabric electrode with high elasticity and low resistance. The smart clothing embeds the sensing of ECG, respiration, acceleration, and gyro information, which intrinsically provides respiration estimations in form of EIP, RIFV, RIAV, RIIV, and respiratory induced movement variation (RIMV). We analyze these five components and combine them by three data fusion techniques (Naïve Bayes inference, static Kalman filter, and dynamic Kalman filter) for robust and accurate estimations. The experimental result shows that the Naïve Bayes inference and the dynamic Kalman filter have the good results.

## Materials and Methods

### Multi-sensor Smart Clothing

The wearable multi-sensor system is designed as a light-weighted and long-term monitoring device. It can be installed on the front chest of the smart clothing, and the user can connect it with cloth by buttons. In addition, through its associated smart phone application, the user can read the ECG, the respiration signal, the activity level, and the temperature in real time.

#### Architecture of Multi-sensor System

The device integrates 24-bit ADC ADS1292R (TI, Dallas, TX, USA), gyroscope & accelerometer MPU-6050 (InvenSense Inc., Sunnyvale, CA, USA), and temperature sensor LMT87D1 (TI, Dallas, TX, USA). The 24-bit ADC ADS1292R is connected to a set of textile electrodes to sense the ECG and respiration signals. The 16-bit gyroscope & accelerometer MPU-6050 can detect 3-axial acceleration and 3-axial angular velocity in movement and respiration, and the temperature sensor LMT87D1 measures the temperature between the smart clothing and coat. The control module also includes a low energy Bluetooth module for wireless transmission and the control module is driven by a small 2032 button battery.

#### Fabric Electrodes

Fabric electrodes are sensing elements fabricated by knitting or weaving processes. Because of its soft and compliant characteristics, they become promising candidates for smart textiles. The traditional textile yarns plated with silver, despite with great conductivity, are known to possess bio-toxic Ag nanoparticles. In [11], they solved this problem by avoiding direct skin contact and they create the non-contact sensors for long-term monitoring. In [33], they stressed the importance of electrodes being skin-friendly for long-time ECG monitoring and developed an embroidered textile electrode by Ag/Ti-coated PET yarn for silver passivation. For the same purpose, Taiwan Textile Research Institute (TTRI) developed non-toxic CB/PBT/PET fibers by conjugate spinning process [34], with a core of polyethylene terephthalate (PET) polymer and a sheath of Carbon Black/polybutylene terephthalate (CB/PBT) polymer. However, CB/PBT/PET yarn has worse electric conductivity than textile yarns plated with silver.

To reduce bio-toxicity and maintain the same grade of electric performance, a new fabric electrode is designed by wrapping the non-toxic CB/PBT/PET yarns around AGposs yarn, the textile yarns plated with silver. We call this new fabric CB/PBT/PET-wrapped AGposs yarn. Because it contacts skins with CB/PBT/PET yarns, it has reduced bio-toxicity and conductivity similar to AGposs yarn.

In order to determine the ideal combination of CB/PBT/PET and AGposs yarns for ECG and respiration measurement, three types of fabric electrodes are experimented: Type 1 electrode of AGposs yarn, Type 2 electrode of CB/PBT/PET-wrapped AGposs yarn (Z twist and 300 turns per meter (TPM)), and Type 3 electrode of CB/PBT/PET-doubly-wrapped AGposs yarn (Z twist and 300 TPM). These electrodes were made by circular knitting machine, Fiber Analysis Knitter-Sampler, Lawson Hemphill Inc., in which the cylinder size is 220 and the needles per inch is 20.

The resistance of a 10-cm fabric electrode was measured under three different conditions:

1. Direct measurement of the resistance without any pre-processing.
2. Measurement of the resistance after soaking the fabric electrode into acid liquid for 24 h and drying it in shadow.
3. Measurement of the resistance after soaking the fabric electrode into alkali liquid for 24 h and drying it in shadow.

According to ISO105-E04, the acid and alkaline solutions were prepared as below:

1. Acid solution, freshly prepared, using grade 3 water complying with ISO 3696, containing, per liter:
  0.5 g of l-histidine monohydrochloride monohydrate (C_6_H_9_O_2_N_3_ **·**HCl**·**H_2_O);
    5 g of sodium chloride (NaCl);
    2.2 g of sodium dihydrogen orthophosphate dihydrate (NaH_2_PO_4_ **·**2H_2_O);
  The solution was brought to pH 5.5 (±0.2) with 0.1 mol/L sodium hydroxide solution.
2. Alkaline solution, freshly prepared, using grade 3 water complying with ISO 3696, containing, per liter:
  0.5 g of l-histidine monohydrochloride monohydrate (C_6_H_9_O_2_N_3_ **·**HCl**·**H_2_O);
    5 g of sodium chloride (NaCl);
  The solution was brought to pH 8 (±0.2) with 0.1 mol/L sodium hydroxide solution.

#### User Interface of Smart Clothing

**Fig. 1:**
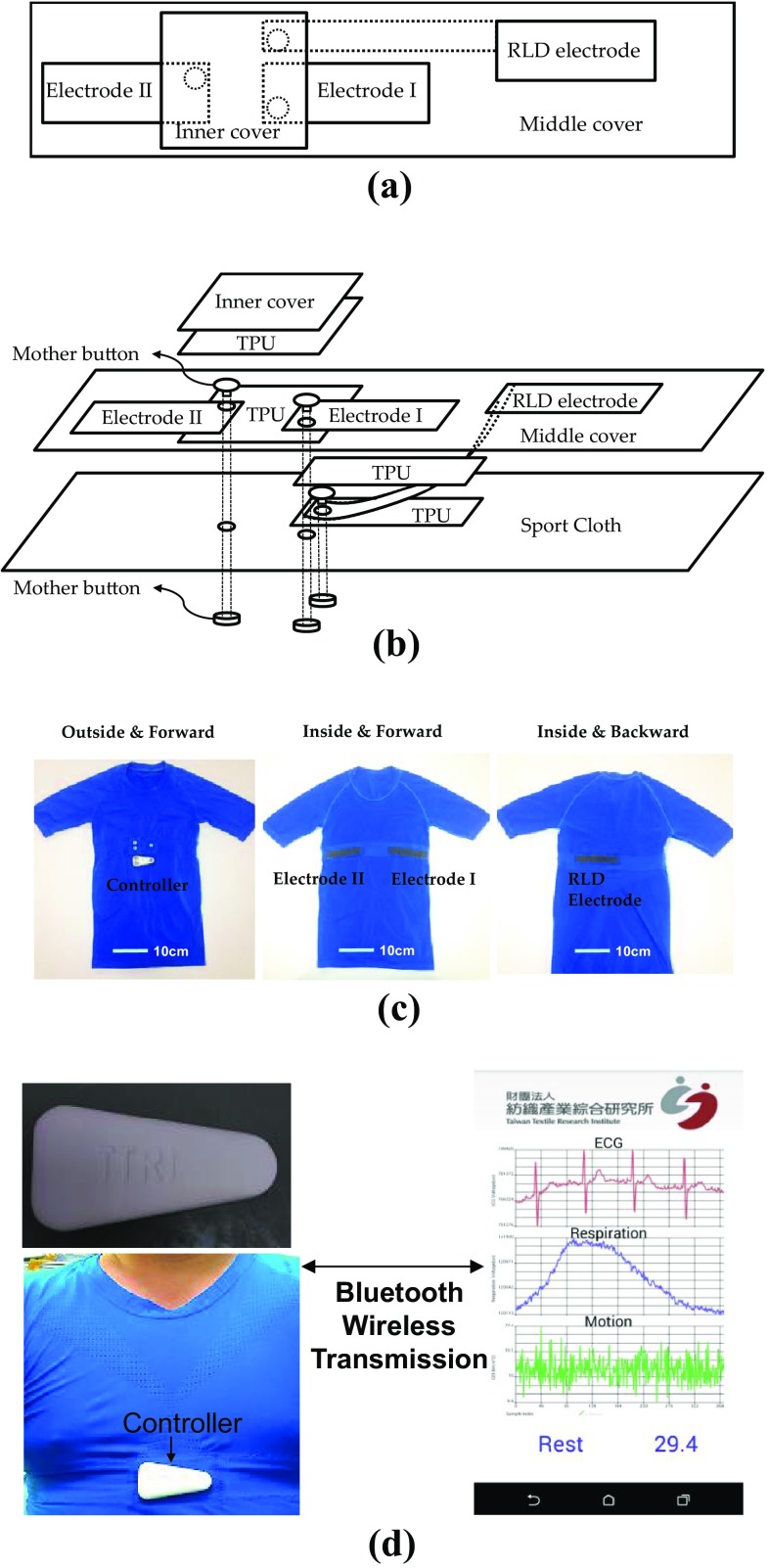
Configuration of the fabric electrodes and the human interface: **a** inside view of the designed placement of electrodes. **b** Exploded view of the designed placement of electrodes. **c** The smart clothing. **d** User interface for monitoring ECG, respiration, acceleration, temperature, and activity level

The designed placement of electrodes is shown in Fig. 1a. The fabric electrodes around the chest consist of right leg driven (RLD) electrode, electrode I, and electrode II. The implementation of RLD loop can improve the common-mode rejection (CMR) in ECG measurement and the electrode I and electrode II are used to measure the ECG and respiratory impedance signal.

The assembly of the textile electrodes is illustrated in Fig. 1b. To avoid potential short circuit between buttons due to sweat, the area between the buttons of electrode 1 and electrode 2 are covered by two paired thermoplastic polyurethane (TPU). To improve the CMR, the RLD electrode is normally placed distant from the other two electrodes and is connected to RLD button by *CB/PBT/PET*-*wrapped AGposs* textile. To avoid the short circuit between textile connector and electrode I, an additional paired TPU is used to cover the connector and the RLD button.

The implemented smart cloth is shown in Fig. 1c. For the elasticity of cloth, the sport cloth is made by knitting structure, and the sport cloth, middle cover, inner cover, and the textile electrodes are combined by zig-zag sewing. The sealing of the paired TPU is made by the 130 °C heated plates. Figure 1d shows the overall of the smart clothing and user interface. The electric signals are transmitted from the fabric-based sensor to the Bluetooth controller through metallic buttons. The smart clothing is soft, elastic, and fitted. The ECG, the respiration, the motion signal, the temperature, and the activity level can be displayed on the screen of a smart phone through Bluetooth wireless transmission.

The electrode’s electric performance was validated by analyzing the signal quality of the fabric electrodes within 0.1–10,000 Hz, where the frequency response is calculated by Eq. (1). The measurement device was NI-USB6218 and we simply test the electric performance by two electrodes without the RLD electrode. The tests were done by putting the smart cloth on a static mannequin to simulate the body wearing situation.

1 $$H\left( s \right) = \frac{{V_{out} \left( s \right)}}{{V_{in} \left( s \right)}}$$

### Respiratory Rate Estimation

The EIP, ECG, acceleration, and angular velocity are collected to compute the five respiratory signals EIP, RIFV, RIAV, RIIV, and RIMV. These signals are processed by peak detection to provide estimates of respiratory rate, and data fusion algorithms are applied to combine them to achieve better estimation.

#### Respiration Signal from EIP

A 0.1–0.8 Hz 2nd-order bandpass Butterworth is applied to extract clean EIP signals for respiratory estimation, because the impedance measurement is affected by ECG, EMG signals and motion artifacts.

#### Respiration Signal from RIFV by Heart Rate Variance

The RIFV signal is extracted using the following three steps:

1. The raw ECG measurement is passed through a 60 Hz 2nd-order band-stop Butterworth filter and a 0.1–55 Hz 2nd-order bandpass filter.
2. The heart rate is calculated by peak detection.
3. A zero-order hold and a 0.1–0.8 Hz 2nd-order bandpass Butterworth filter are applied to smooth the signal of heart rate variation and the RIFV signal is extracted finally for respiratory rate estimation.

#### Respiration Signal from RIAV by Baseline Removal and Kurtosis

In [35], the feature of RIAV is extracted by kurtosis, and the accuracy is above 93.48%. Here, we modify the kurtosis methods as described below. Firstly, the baseline-free ECG signal is extracted from the raw ECG measurement by a 60 Hz 2nd-order band-stop Butterworth filter and a 10–55 Hz 2nd-order bandpass Butterworth filter. Secondly, R peak detection is applied to extract the data between two R peaks. Thirdly, these data between two R peaks is used to calculate the kurtosis as Eq. (2) and the normalized kurtosis as Eq. (3) is calculated by the mean of kurtosis and standard deviation of kurtosis *σ*_*K*_.

2 $$Kurtosis{:}\; K\left( i \right) = E\left\{ {x\left( n \right)^{4} } \right\}_{{n_{i} \le n < n_{i + 1} }} - 3\left( {E\left\{ {x\left( n \right)^{2} } \right\}_{{n_{i} \le n < n_{i + 1} }} } \right)^{2}$$

3 $$Normalized\;Kurtosis{:}\;K_{normalized} \left( i \right) = \frac{{\left[ {K\left( i \right) - E\left\{ K \right\}} \right]}}{{\sigma_{K} }}$$

where *i* denotes the *i*th R–R interval, *K*(*i*) denotes the *i*th kurtosis. The time of *K*(*i*) is defined as the middle of the time of the *n*_*i*_th and the *n*_*i*+1_th R peaks. After the kurtosis calculation, linear interpolation is applied to smooth the result and a 0.1–0.8 Hz 2nd-order bandpass Butterworth filter is used to generate respiratory signal from RIAV.

#### Respiration Signal from RIIV by ECG Signal Removal

The baseline signal in raw ECG is extracted as the RIIV signal. In processing, a 60 Hz 2nd-order band-stop Butterworth filter is used to remove the 60 Hz noise and a 0.1–0.8 Hz 2nd-order bandpass Butterworth filter is applied to extract the respiratory rate estimate from the ECG signal.

#### Respiration Signal from Euler Angle by Fusing Accelerometer and Gyroscope

**Fig. 2:**
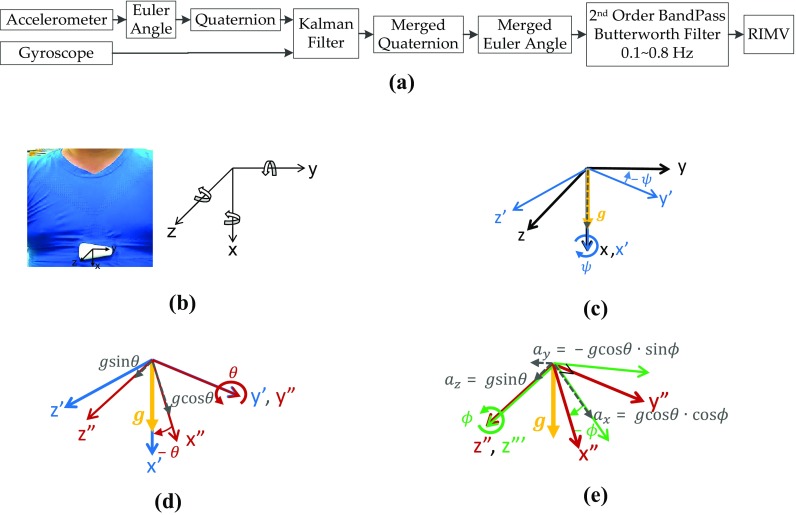
**a** The signal processing of RIMV. **b** The coordination of body frame. **c** The rotation of *x-axis* in body coordination. **d** The rotation of *y’-axis* in body coordination. **e** The rotation of *z″-axis* in body coordination

For RIMV, the 3-axial acceleration and the 3-axial angular velocity are merged to calculate the quaternion by Kalman filter [25, 36], as shown in Fig. 2a. We define the acceleration and angular velocity in the body coordination system shown in Fig. 2, in which the x-axis is defined to be aligned with the gravity force and z-axis is chosen to be pointing forward. The Euler angles are defined as a set of three rotation angles $\left( {\psi , \theta ,\phi } \right)$ about x-, y’-, z”-axes. Because respiration causes mostly the change in *θ* about y-axis in the body coordination, the variation of *θ* is used as RIMV signal, which can be derived as follows. For a fixed gravity vector, the rotation of body coordination about x-, y-, z-axes with respect to body coordination can be derived as

4 $$R_{x} \left( \psi \right) = \left[ {\begin{array}{*{20}c} 1 & 0 & 0 \\ 0 & {\cos \psi } & {\sin \psi } \\ 0 & { - \sin \psi } & {\cos \psi } \\ \end{array} } \right]$$

5 $$R_{y} \left( \theta \right) = \left[ {\begin{array}{*{20}c} {\cos \theta } & 0 & { - \sin \theta } \\ 0 & 1 & 0 \\ {\sin \theta } & 0 & {\cos \theta } \\ \end{array} } \right]$$

6 $$R_{z} \left( \phi \right) = \left[ {\begin{array}{*{20}c} {\cos \phi } & {\sin \phi } & 0 \\ { - \sin \phi } & {\cos \phi } & 0 \\ 0 & 0 & 1 \\ \end{array} } \right]$$

Therefore the rotation matrix with respect to body coordination following the xyz rotation sequence is.

7 $$\begin{array}{llll} R_{xyz} = R_{z} \left( \phi \right) \cdot R_{y} \left( \theta \right) \cdot R_{x} \left( \psi \right) = \hfill \\ \left[ {\begin{array}{*{20}c} {\cos \theta \cos \phi } & {\sin \psi \sin \theta \cos \phi + \cos \psi \sin \phi } & { - \cos \psi \sin \theta \cos \phi + \sin \psi \sin \phi } \\ { - \cos \theta \sin \phi } & { - \sin \psi \sin \theta \sin \phi + \cos \psi \cos \phi } & {\cos \psi \sin \theta \sin \phi + \sin \psi \cos \phi } \\ {\sin \theta } & { - \sin \psi \cos \theta } & {\cos \psi \cos \theta } \\ \end{array} } \right] \hfill \\ \end{array}$$

If the movement is slow, then the accelerometer measurement in body coordination can be approximated as

8 $$\left[ {\begin{array}{*{20}c} {a_{x} } \\ {a_{y} } \\ {a_{z} } \\ \end{array} } \right] \cong R_{xyz} \cdot \left[ {\begin{array}{*{20}c} g \\ 0 \\ 0 \\ \end{array} } \right] = \left[ { - \begin{array}{*{20}c} {\cos \theta \cos \phi \cdot g} \\ {\cos \theta \sin \phi \cdot g} \\ {\sin \theta \cdot g} \\ \end{array} } \right]$$

The Euler angle $\theta {\text{\;and }}\phi$ could be derived by Eqs. (9) and (10) through accelerometer measurement $\left( {a_{x} , a_{y} , a_{z} } \right)$. Because the respiration movement is mainly due to change in *θ*, *ψ* is not needed. On the other hand, *ψ* cannot be calculated with the gravity vector; therefore, *ψ* is set as zero here. In some applications, the three Euler angles may be needed. In this situation, a magnetometer should be included to derive the three Euler angles:

9 $$\theta = \sin^{ - 1} \left( {\frac{{a_{z} }}{\text{g}}} \right)$$

10 $$\phi = \tan^{ - 1} \left( {\frac{{ - a_{y} }}{{a_{x} }}} \right) = \sin^{ - 1} \left( {\frac{{ - a_{y} }}{{{\text{gcos}}\theta }}} \right)$$

The Euler angle estimation using accelerometer is noisy; to increase the accuracy, we also use the angular velocity derived from gyroscope. We use Kalman filter based on quaternions to merge these two estimates. Note that we formulate the system equations in quaternions, so the singularity issue in Euler angle representation can be avoided.

The Kalman filter algorithm for quaternions is summarized as below.

*Time Update*

11 $$\hat{x}_{q,k}^{ - } = A\hat{x}_{q,k - 1}$$

12 $$P_{q,k}^{ - } = P_{q,k - 1} + Q_{q}$$

13 $$A = I + \frac{1}{2} \cdot \Delta t \cdot \left[ {\begin{array}{*{20}c} 0 & {\omega_{x,k - 1} } & {\omega_{y,k - 1} } & {\omega_{z,k - 1} } \\ { - \omega_{x,k - 1} } & 0 & { - \omega_{z,k - 1} } & {\omega_{y,k - 1} } \\ { - \omega_{y,k - 1} } & {\omega_{z,k - 1} } & 0 & { - \omega_{x,k - 1} } \\ { - \omega_{z,k - 1} } & { - \omega_{y,k - 1} } & {\omega_{x,k - 1} } & 0 \\ \end{array} } \right]$$

14 $$Q_{q} = 10^{ - 4} \cdot I_{4} , P_{q,0}^{ - } = I_{4} , \hat{x}_{q,0} = z_{q,0}$$

Here, $\omega_{x,k - 1} , \omega_{y,k - 1} , {\text{and\;}} \omega_{z,k - 1}$ are the angular velocities of x-, y-, z-axes of the body coordination at the time *t*_*k*-1_. The gyroscope measures the angular velocity in body coordinate; therefore, the angular velocities of a fixed gravity vector with respect to body coordination are $- \omega_{x,k - 1} , - \omega_{y,k - 1} , {\text{and}} - \omega_{z,k - 1}$ and the state update matrix is described by matrix *A.* $\hat{x}_{q,k - 1}^{ - } \in \Re^{4}$ is the prior state estimate of quaternions and $P_{q,k - 1}^{ - } \in \Re^{4 \times 4}$ is the prior error covariance matrix at time *t*_*k*_. $\hat{x}_{q,k - 1}^{ - } \in \Re^{4}$ is the posterior state estimate of quaternions and $P_{q,k - 1} \in \Re^{4 \times 4}$ is the posterior error covariance matrix at time *t*_*k*-1_ given measurement $z_{q,k - 1} \in \Re^{4}$

*Measurement Update*

15 $$K_{q,k} = P_{q,k}^{ - } H_{q}^{T} \left( {H_{q} P_{q,k}^{ - } H_{q}^{T} + R_{q} } \right)^{ - 1}$$

16 $$\hat{x}_{q,k} = \hat{x}_{q,k}^{ - } + K_{q,k} \left( {z_{q,k} - H_{q} \hat{x}_{q,k}^{ - } } \right)$$

17 $$P_{q,k} = P_{q,k}^{ - } - K_{q,k} H_{q} P_{q,k}^{ - }$$

18 $$z_{q,k} = \left[ {\begin{array}{*{20}c} {q_{0} } \\ {q_{1} } \\ {q_{2} } \\ {q_{3} } \\ \end{array} } \right] = \left[ \begin{aligned} \cos \frac{\psi }{2}\cos \frac{\theta }{2}\cos \frac{\phi }{2} - \sin \frac{\psi }{2}\sin \frac{\theta }{2}\sin \frac{\phi }{2} \hfill \\ - \sin \frac{\psi }{2}\sin \frac{\theta }{2}\cos \frac{\phi }{2} - \cos \frac{\psi }{2}\cos \frac{\theta }{2}\sin \frac{\phi }{2} \hfill \\ - \cos \frac{\psi }{2}\sin \frac{\theta }{2}\cos \frac{\phi }{2} + \sin \frac{\psi }{2}\cos \frac{\theta }{2}\sin \frac{\phi }{2} \hfill \\ - \sin \frac{\psi }{2}\cos \frac{\theta }{2}\cos \frac{\phi }{2} - \cos \frac{\psi }{2}\sin \frac{\theta }{2}\sin \frac{\phi }{2} \hfill \\ \end{aligned} \right]$$

The matrix $H_{q} = I_{4} \in^{4 \times 4}$ relates the state $\hat{x}_{q,k}$ to the measurement quaternions *z*_*q*,*k*_. The $K_{q,k} \in \Re^{4 \times 4}$ is the Kalman gain to minimize the posteriori error covariance matrix *P*_*q*,*k*_.

After the Kalman filter, the merged quaternions are translated back into the Euler angles by Eq. (19) and the Euler angle *θ* is set as the motion signal induced by respiration.

19 $$\left[ {\begin{array}{*{20}c} \psi \\ \theta \\ \phi \\ \end{array} } \right] = \left[ {\begin{array}{*{20}c} {\arctan \frac{{ - 2\left( {q_{0} q_{1} + q_{2} q_{3} } \right)}}{{1 - 2\left( {q_{1}^{2} + q_{2}^{2} } \right)}}} \\ {\arcsin \left( {2\left( {q_{1} q_{3} - q_{0} q_{2} } \right)} \right)} \\ {\arctan \frac{{ - 2\left( {q_{0} q_{3} + q_{1} q_{2} } \right)}}{{1 - 2\left( {q_{2}^{2} + q_{3}^{2} } \right)}}} \\ \end{array} } \right]$$

Finally, through a 2nd-order bandpass Butterworth filter, the smooth motion signal is treated as the RIMV estimate.

### Data Fusion for Respiratory Rate Estimation

There are two hypotheses assumed in three data fusion methods:

1. The respiratory rate changes slowly in normal respiration.
2. The more accurate a respiration estimation method is, the smaller variance in respiration estimation it has.

#### Fusion Method 1: Naïve Bayes Inference

In order to merge the five respiratory rate estimates, we firstly adopt Naïve Bayes inference. In this methods, the less reliable estimates will have larger variances *σ*_*i*, *k*_^2^ and small weights 1/*σ*_*i*, *k*_^2^. The fusion equation is shown in Eq. (20), in which *z*_*i*,*k*_ are the five respiratory rate estimates at time instance *t*_*k*_, the variances *σ*_*i*, *k*_^2^ are calculated by the measuring the respiratory rate in the window of past 30 s, and the *μ*_*k*_ is the estimated respiratory rate by Naïve Bayes inference.

20 $$\mu_{k} = \frac{{\mathop \sum \nolimits_{i = 1}^{5} \frac{{z_{i,k} }}{{\sigma_{i,k}^{2} }}}}{{\mathop \sum \nolimits_{i = 1}^{5} \frac{1}{{\sigma_{i,k}^{2} }}}}$$

#### Fusion Method 2: Static Kalman Filter Data Fusion

Because the respiratory rate does not change rapidly, the state-space model for respiratory rate can be approximated as Eq. (21) and Eq. (22). *x*_*k*_ is the estimate of the respiratory rate and *w*_*k*_ is the process noise.$Z_{k} \in \Re^{5}$ is the measured respiratory rate and $V_{k} \in \Re^{5}$ is the measurement noise in EIP(*z*_1_), RIFV(*z*_2_), RIAV(*z*_3_), RIIV(*z*_4_), and RIMV(*z*_5_).

21 $$x_{k} = x_{k - 1} + w_{k}$$

22 $$Z_{k} = Hx_{k} + V_{k}$$

23 $$Z_{k} = \left[ {z_{1} z_{2} z_{3} z_{4} z_{5} } \right]^{T} , H = I_{5 \times 1} \in \Re^{5 \times 1}$$

Here, *w*_*k*_ and *V*_*k*_ are assumed to be white, independent and have normal probability distribution.

24 $$p\left( {w_{k} } \right) \sim N\left( {0, Q} \right)$$

25 $$p\left( {V_{k} } \right) \sim N\left( {0, R} \right)$$

$Q \in \Re^{1}$ is the process noise covariance and $R \in \Re^{5 \times 5}$ is the measurement noise covariance matrix. Though they might change with time, here *Q* = 1 is set as constant value and the noise covariance matrix *R* is empirically set as $R_{s} \in \Re^{5 \times 5}$, where

26 $$R = R_{s} = 100 \cdot {\text{I}}_{5}$$

The procedure for Kalman filter of respiratory rate estimation is to first predict the state variable under the last state estimation in the time-update cycle and adjust the state variable according the five measured respiratory rates in the measurement-update cycle.

*Time Update*

27 $$\hat{x}_{k}^{ - } = \hat{x}_{k - 1}$$

28 $$p_{k}^{ - } = p_{k - 1} + q$$

*Measurement Update*

29 $$K_{k} = P_{k}^{ - } H^{T} \left( {HP_{k}^{ - } H^{T} + R} \right)^{ - 1}$$

30 $$\hat{x}_{\text{k}} = \hat{x}_{k}^{ - } + K_{k} \left( {Z_{k} - H\hat{x}_{k}^{ - } } \right)$$

31 $$p_{k} = p_{k}^{ - } - K_{k} Hp_{k}^{ - }$$

Here, $\hat{x}_{k}^{ - } \in \Re^{1}$ is the prior state estimate of respiratory rate at time point *t*_*k*_ and $\hat{x}_{k}$ is the posterior state estimate of respiratory rate at time point *t*_*k*_ given measurement respiratory rate $Z_{k} \in \Re^{5}$. $p_{k}^{ - } \in \Re^{1}$ is the prior error variance and $p_{k} \in \Re^{1}$ is the posteriori error variance. The matrix *K*_*k*_ is the Kalman gain to minimize the posterior error variance *p*_*k*_. The initial parameter is set as $\hat{x}_{0} = 0,\;p_{0} = 1$.

#### Fusion Method 3: Dynamic Kalman Filter Data Fusion

Here dynamic measurement noise covariance matrix $R_{d} \in \Re^{5 \times 5}$ in Eq. (32) is used. The variance *σ*_*i*, *k*_^2^ is calculated by the measured respiratory rate in the window of the past 30 s. Through the dynamic change of the measurement noise covariance matrix, the dynamic Kalman filter will fuse the data adaptively according the recently observations.

32 $$R = R_{d} = \left[ {\begin{array}{*{20}c} {\sigma_{1,k}^{2} } & 0 & 0 & 0 & 0 \\ 0 & {\sigma_{2,k}^{2} } & 0 & 0 & 0 \\ 0 & 0 & {\sigma_{3,k}^{2} } & 0 & 0 \\ 0 & 0 & 0 & {\sigma_{4,k}^{2} } & 0 \\ 0 & 0 & 0 & 0 & {\sigma_{5,k}^{2} } \\ \end{array} } \right]$$

### Experiments

The experimental procedure for static sitting is composed of six tests of respiratory rates 8, 25, 10, 20, 12, 15 rcpm. Fifteen subjects participated and were instructed to sit and follow the metronome to breath. Between the tests, the subjects had 30 s to move their body and relax. Signals from EIP, accelerometer, gyroscope, and ECG were acquired in 250 Hz sample rate.

For the dynamic running experiment, the running speed first increases from 3 km/h to 9 km/h and decreases from 9 km/h back to 3 km/h. The fifteen subjects were instructed to run while wearing the nose tube and the respiration signal was measured by capnography (MD-800, COMDEK Industrial Corp). Signals from EIP, accelerometer, gyro, and ECG were acquired in 250 Hz and the capnography was sampled in 50 Hz. The capnography was used to generate the standard respiratory rate as the ground truth to evaluate the estimated respiratory rate in the running task.

## Results and Discussion

### Results of Electrodes Resistance

Table 2 summarizes the experimental results of resistance tests. The resistance of Type 2 electrode (CB/PBT/PET-wrapped AGposs yarn) is better than Type 3 electrode (CB/PBT/PET-doubly- wrapped AGposs yarn), and similar to Type 1 electrode (AGposs yarn). Therefore, Type 2 fabric electrode is chosen for the smart clothing.

**Table 2:** The resistance of electrodes made of three types of conductive fabric under three conditions

Fabric type	Type 1	Type 2	Type 3
Wrapped yarn		CB/PBT/PET yarn 70/24f	Double CB/PBT/PET yarn 70/24f
Components of wrapped yarn	AGposs 70d/34f	AGposs 70d/34f; 300TPM/Z Twist	AGposs 70d/34f; 300TPM/Z Twist
Textile electrode			
Fabric without any process	1.2 ± 0.1 Ω	2.3 ± 0.2 Ω	8.3 ± 1.2 Ω
Acid liquid	14.7 ± 2.1 Ω	9.9 ± 2.1 Ω	14.7 ± 2.1 Ω
Alkali liquid	6.5 ± 0.8 Ω	5.8 ± 0.1 Ω	6.5 ± 0.8 Ω

*Type 1* electrode made of AGposs yarn

*Type 2* electrode made of CB/PBT/PET-wrapped AGposs yarn

*Type 3* electrode made of CB/PBT/PET-doubly-wrapped AGposs yarn

### Results of Electrodes Impedance

**Fig. 3:**
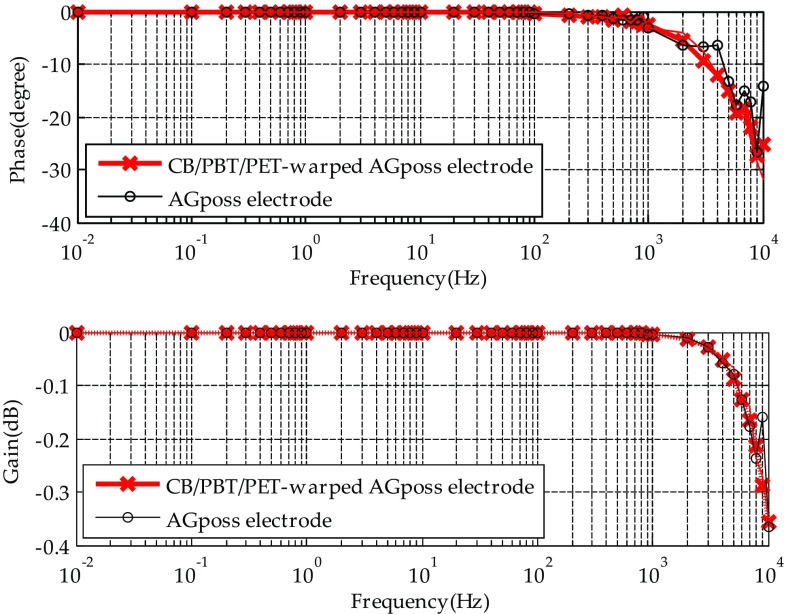
The frequency response of fabric electrodes

Figure 3 shows the experimental result. Type 1 and Type 2 electrodes can pass signals in the range of 0.01–10,000 Hz, and both of them have similar bandwidth. Though the phase delay becomes significant for signals over 1000 Hz, the proposed fabric electrodes are still adequate for ECG sensing, because the bandwidth of ECG signal is only about 0.1–55 Hz.

### Examples of Respiratory Waveform

For illustration, this section shows the five respiratory signals extracted from the EIP, RIFV, RIAV, RIIV, and RIMV in the 12-rcpm testing of subject 12 (s12).

#### Respiration Signal from EIP

**Fig. 4:**
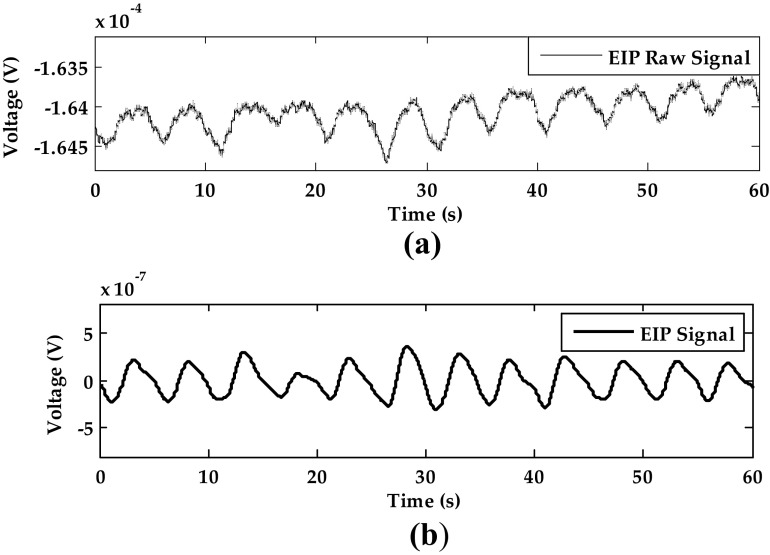
The EIP signal processing of subject 12 in static sitting

The raw signal of EIP is shown in Fig. 4a and the filtered EIP signal is shown in Fig. 4b. After the signal processing, the EIP signal is smooth and near zero mean. The time interval between two peaks is used to calculate the respiratory rate.

#### Respiration Signal from RIFV by Heart Rate Variance

**Fig. 5:**
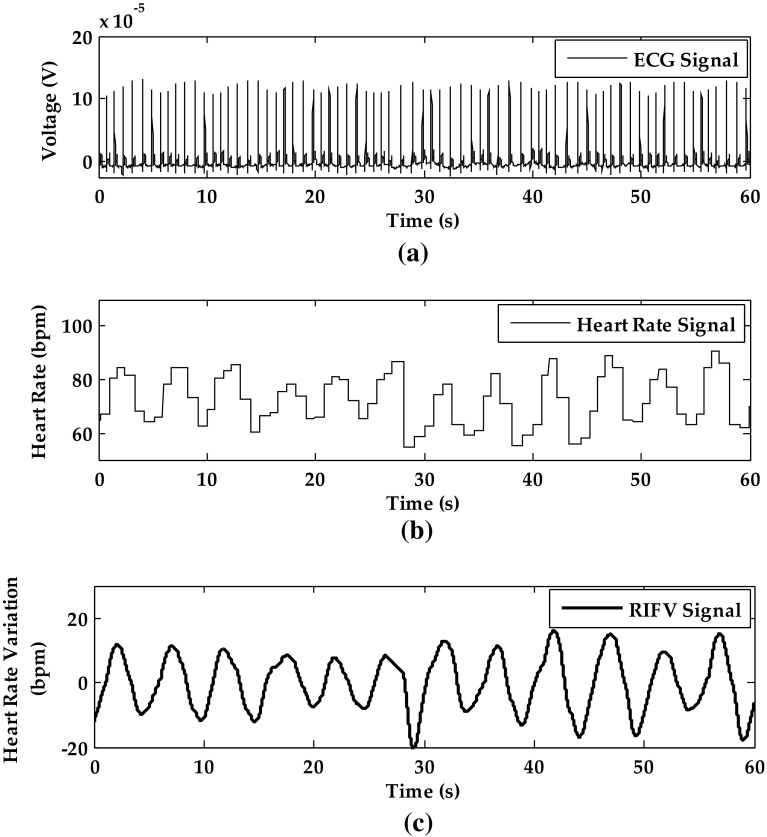
The RIFV signal processing of subject 12 in static sitting

The ECG is shown in Fig. 5a and the heart rate signal is calculated by R-peak detection, as shown in Fig. 5b. Through the zero-order hold and filtering, the smooth RIFV signal shown in Fig. 5c is clear for respiratory rate calculation.

#### Respiration Signal from RIAV by Baseline Removal and Kurtosis

**Fig. 6:**
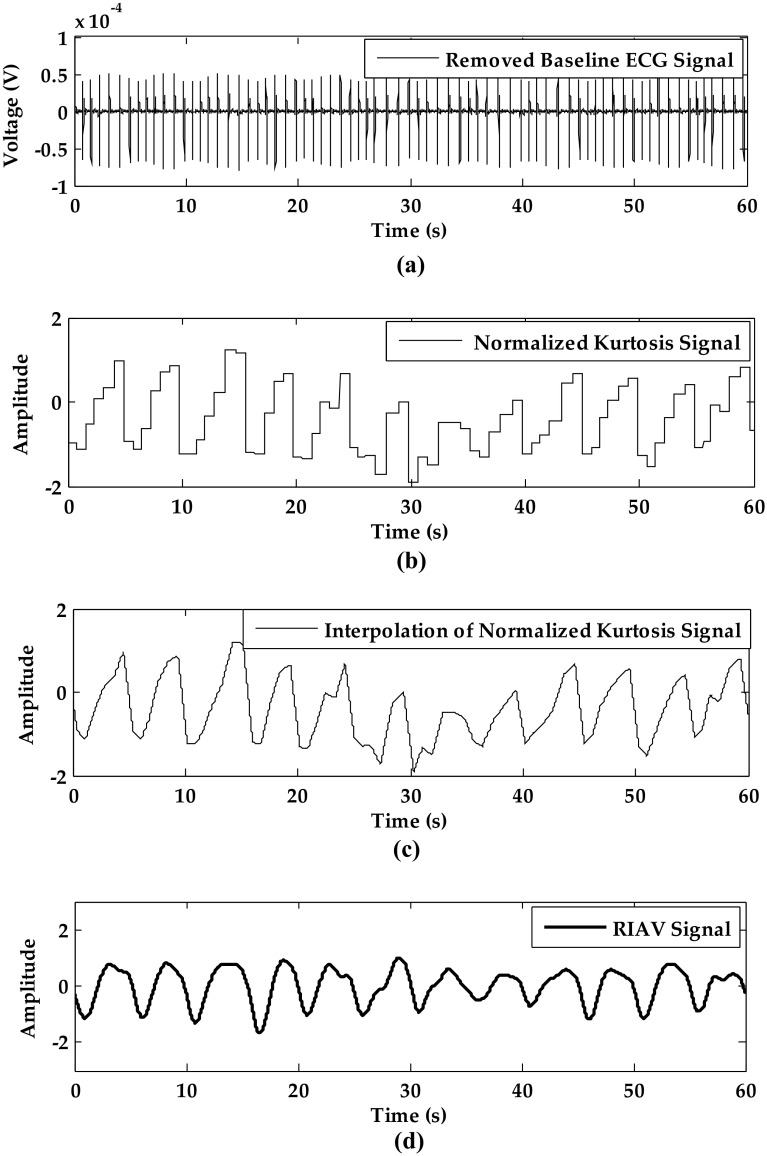
The RIAV signal processing of subject 12 in static sitting

The baseline free ECG signal is shown in Fig. 6a and the kurtosis between R-peak is shown in Fig. 6b. Through the linear interpolation, the smoothed kurtosis signal is shown in Fig. 6c. Finally, the RIAV signal generated by filtering is shown in Fig. 6d.

#### Respiration Signal from RIIV by ECG Signal Removal

**Fig. 7:**
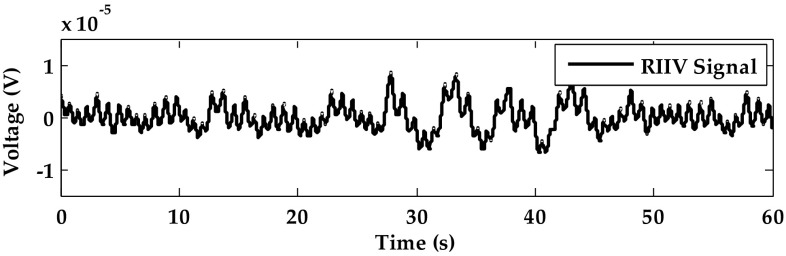
The RIIV signal processing of subject 12 in static sitting

After removing the ECG signal, the baseline signal (RIIV) is shown in Fig. 7. The respiratory signal from RIIV is not clear for subject 12.

#### Respiration Signal from Motion by Fusing Accelerometer and Gyroscope

**Fig. 8:**
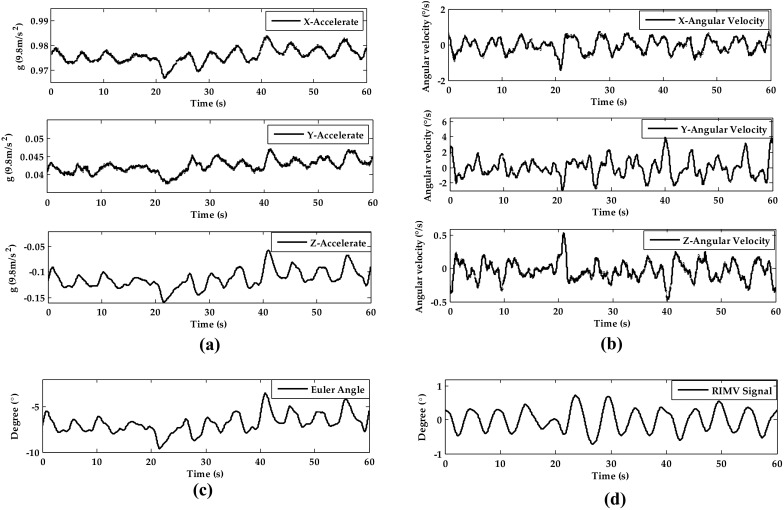
The signal processing of RIMV of subject 12 in static sitting

The three accelerations and angular velocities are shown in Fig. 8a and Fig. 8b. The calculated Euler angle *θ* is shown in Fig. 8c and the filtered respiratory signal from RIMV is shown in Fig. 8d. The respiratory signal here is clear enough to estimate the respiratory rate.

### Respiratory Rate Estimation

**Fig. 9:**
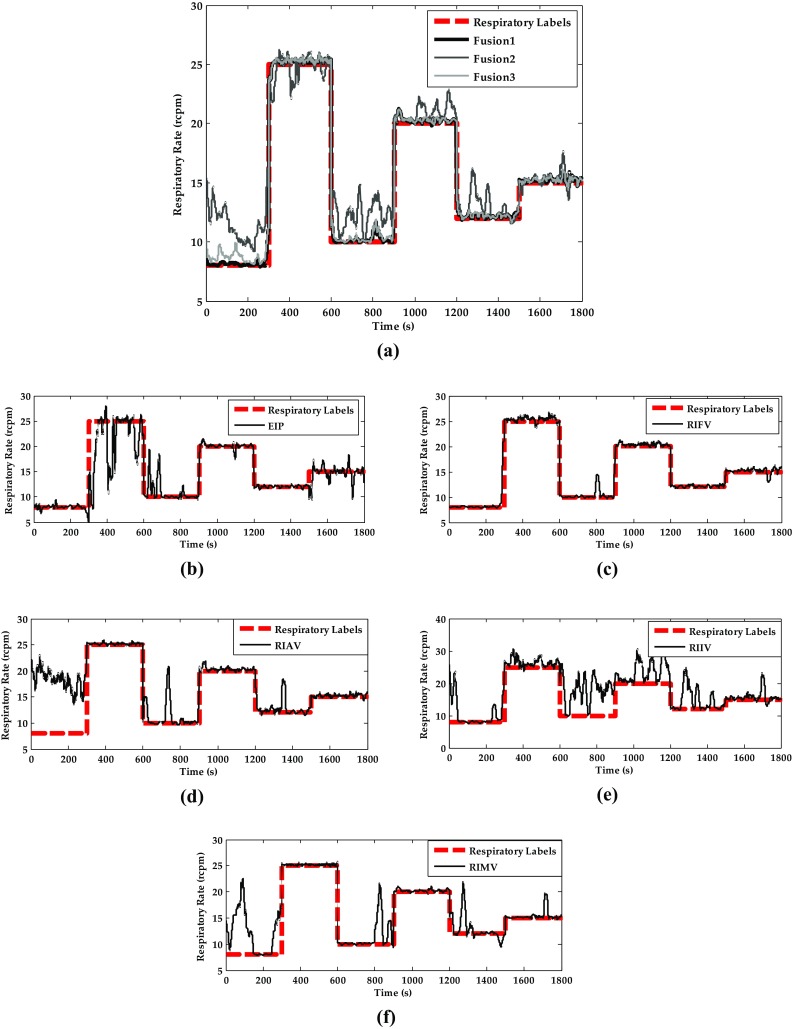
The estimated respiratory rates of subject 12 across six tests in static sitting. **a** Respiratory estimation of the three fusion methods. **b** Respiratory estimation of EIP. **c** Respiratory estimation of RIFV. **d** Respiratory estimation of RIAV. **e** Respiratory estimation of RIIV. **f** Respiratory estimation of RIMV

After the respiratory signals are generated, peak detection is applied to calculate the respiratory rate. The estimated respiratory rates of the eight methods are shown in Fig. 9. Here, Fusion 1 is Naïve Bayes inference, Fusion 2 is static Kalman filter, and Fusion 3 is dynamic Kalman filter. We observe that the estimated respiratory rates are distributed close to the target respiratory rate but with different variance in the static experiment. Although the respiratory rates are inaccurate in some cases, especially in RIIV, Fusion 1 and Fusion 3, which merges the respiratory rates from EIP, RIFV, RIAV, RIIV, and RIMV, have consistently high accuracy in respiratory rate estimation across all six tests.

### The Accuracy of Respiratory Rate Estimation in Static Sitting

The mean and the standard deviation of absolute respiratory rate error are shown in Table 3. For the best result (s12), the minimum mean of absolute error in Fusion 1 is 0.3 rcpm. The worst result is 7.9 rcpm in RIIV of s11, of which the mean of absolute error in Fusion 1 is 0.6 rcpm and the mean of absolute error in Fusion 3 is 0.8 rcpm. For all subjects, Fusion 1 and Fusion 3 have the lowest mean of absolute error and similar performance. But it also reveals that the means by fusing a very good signal RIMV (mean of absolute error: 0.6 rcpm) with the other four signals, the fusion may yield worse results (mean of absolute error in Fusion 1: 0.7 rcpm) than utilizing the single best sensor alone, such as s8. As for the five respiratory signals, RIFV has the best performance (mean of all subjects: 1.4 rcpm), the second is RIMV (mean of all subjects: 1.8 rcpm), the third is RIAV (mean of all subjects: 2.3 rcpm), the forth is EIP (mean of all subjects: 2.9 rcpm), and the fifth is RIIV (mean of all subjects: 3.9 rcpm).

**Table 3:** The mean (standard deviation) of absolute respiratory rate error over 6 tests in static sitting

Subject	Fusion 1	Fusion 2	Fusion 3	EIP	RIFV	RIAV	RIIV	RIMV
s1	0.4 (0.6)	1.8 (1.4)	0.6 (0.7)	1.5 (2.6)	1.1 (1.9)	4.3 (3.5)	0.6 (1.0)	3.1 (4.0)
s2	0.8 (1.6)	1.9 (1.6)	0.9 (1.5)	3.5 (4.8)	0.9 (1.6)	1.7 (2.3)	4.1 (4.0)	1.2 (1.8)
s3	0.4 (0.5)	1.8 (1.4)	0.4 (0.6)	3.7 (4.4)	0.7 (1.0)	4.2 (3.8)	2.0 (2.8)	0.7 (1.2)
s4	0.7 (1.6)	1.8 (1.5)	0.8 (1.5)	1.9 (3.8)	0.8 (1.7)	2.4 (3.3)	3.9 (3.7)	1.1 (2.2)
s5	0.6 (0.9)	1.9 (1.5)	0.6 (0.9)	2.6 (3.9)	1.1 (1.8)	1.5 (2.4)	4.7 (4.2)	1.7 (2.3)
s6	0.4 (0.7)	1.3 (1.2)	0.5 (0.7)	2.3 (2.9)	0.7 (1.4)	2.9 (3.1)	1.3 (2.1)	0.7 (1.2)
s7	1.3 (1.2)	2.2 (1.6)	1.4 (1.2)	3.7 (4.2)	3.1 (3.4)	2.1 (2.2)	3.0 (2.9)	2.2 (2.5)
s8	0.7 (1.1)	2.8 (1.7)	0.9 (1.1)	3.9 (4.6)	1.5 (2.2)	5.2 (3.6)	4.6 (3.6)	0.6 (1.3)
s9	0.7 (1.0)	1.9 (1.6)	0.8 (1.0)	4.6 (5.3)	1.1 (1.8)	1.2 (1.9)	3.0 (3.4)	1.9 (2.5)
s10	0.4 (0.4)	1.5 (1.2)	0.4 (0.4)	3.1 (4.7)	1.1 (1.8)	1.1 (1.8)	3.4 (3.5)	1.0 (2.0)
s11	0.6 (1.2)	3.2 (1.8)	0.8 (1.2)	1.8 (3.5)	3.6 (4.0)	2.1 (3.6)	7.9 (5.2)**	2.8 (3.2)
s12	0.3 (0.3)*	1.6 (1.5)	0.4 (0.4)	1.2 (2.5)	0.5 (1.1)	2.3 (3.9)	3.3 (3.9)	1.5 (2.8)
s13	0.4 (0.6)	2.0 (1.6)	0.6 (0.7)	1.6 (2.2)	0.6 (1.3)	0.5 (1.2)	6.0 (4.9)	2.7 (3.1)
s14	0.9 (1.1)	2.0 (1.4)	1.0 (1.1)	4.5 (4.3)	1.1 (1.5)	1.4 (2.1)	3.5 (3.5)	2.4 (2.4)
s15	1.9 (2.0)	3.5 (2.1)	2.0 (1.9)	4.4 (4.8)	3.0 (3.1)	1.5 (1.7)	6.6 (4.1)	4.1 (3.8)
Mean	0.7 (1.0)	2.1 (1.5)	0.8 (1.0)	2.9 (3.9)	1.4 (2.0)	2.3 (2.7)	3.9 (3.5)	1.8 (2.4)

*EIP* electric impedance plethysmography; *RIAV* respiratory induced amplitude variation; *RIFV* respiratory induced frequency variation; *RIIV* respiratory induced intensity variation; *RIMV* respiratory induced movement variation

* The best result

** The worst result

**Fig. 10:**
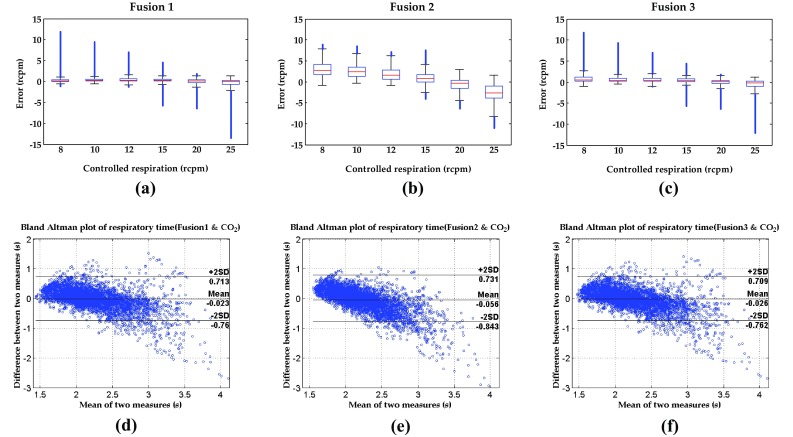
**a**–**c** The box plots for three fusion algorithms over all subjects in controlled respiration. **d**–**f** The Bland–Altman plots of respiratory time for three fusion algorithms over 15 subjects during running

In order to see if the fusion performance is better in some specific respiratory rates, the box plot for three fusion algorithms in static task over all subjects are applied, as shown in Fig. 10a–c. The X-axis is the controlled respiratory rate and the Y-axis is the respiratory rate errors between the controlled respiratory rate and the fusion method. Comparisons of three fusion methods, Fusion 1 and Fusion 3 have better results than Fusion 2. The median error is near zero and the values of the Q1–1.5 IQR (interquartile range) and the Q1 + 1.5 IQR are within 3 to −3 rcpm in Fusion 1 or Fusion 3.

### The Accuracy of Respiratory Rate Estimation During Running

The mean and the standard deviation of absolute respiratory rate error during exercise are shown in Table 4. For the best result (s7), the minimum absolute mean error in Fusion 1 is 1.8 rcpm. The worst result is 10.3 rcpm in RIIV of s3, of which the absolute mean error in Fusion 1 is 4.0 rcpm. The averages of absolute mean errors of all 8 respiratory signals are worse in running than in static sitting. It reveals that dynamic movements affect all the respiratory signals. But the averages of absolute mean errors in Fusion 1 and Fusion 3 are still within 3 rcpm.

**Table 4:** The mean (standard deviation) of absolute respiratory rate error during running

Subject	Fusion 1	Fusion 2	Fusion 3	EIP	RIFV	RIAV	RIIV	RIMV
s1	3.6 (3.4)	3.8 (3.6)	3.6 (3.4)	3.4 (2.7)	4.4 (4.0)	3.9 (3.3)	5.6 (5.0)	7.3 (7.2)
s2	4.0 (3.6)	4.0 (3.1)	4.0 (3.6)	3.7 (2.8)	5.7 (4.2)	5.3 (3.9)	4.6 (3.6)	7.7 (5.5)
s3	4.0 (3.5)	4.9 (4.0)	4.0 (3.5)	4.4 (4.3)	4.5 (4.4)	1.8 (2.0)	10.3 (6.3)**	8.2 (6.6)
s4	3.0 (3.4)	3.9 (3.4)	4.0 (3.4)	4.4 (3.5)	4.7 (3.7)	3.4 (3.1)	4.4 (3.9)	7.3 (5.3)
s5	3.4 (2.7)	4.2 (3.0)	3.4 (2.7)	7.8 (5.1)	4.8 (3.8)	5.7 (3.8)	7.4 (6.3)	3.6 (3.3)
s6	1.9 (2.0)	2.4 (2.0)	2.0 (2.0)	3.0 (2.8)	5.7 (4.3)	4.1 (3.5)	4.3 (3.9)	4.0 (4.8)
s7	1.8 (1.9)*	2.3 (1.8)	1.8 (1.9)	1.6 (1.5)	3.0 (2.9)	2.7 (2.4)	3.3 (3.9)	5.6 (4.5)
s8	3.3 (2.9)	3.5 (2.7)	3.3 (2.8)	4.1 (3.4)	4.2 (3.6)	3.8 (2.9)	4.4 (3.8)	6.1 (5.0)
s9	3.3 (3.1)	4.0 (3.4)	3.3 (3.1)	6.3 (6.2)	4.4 (3.4)	4.8 (3.8)	5.6 (5.9)	2.9 (2.4)
s10	1.9 (1.7)	3.1 (2.0)	1.9 (1.6)	4.5 (3.1)	2.8 (2.3)	3.1 (3.0)	6.2 (4.6)	2.0 (2.5)
s11	3.3 (2.9)	3.6 (3.0)	3.4 (2.8)	3.4 (2.8)	4.8 (4.2)	2.3 (2.3)	5.7(4.8)	7.7 (5.5)
s12	2.7 (1.9)	3.0 (2.1)	2.8 (1.8)	4.0 (3.0)	2.2 (1.8)	4.0 (2.7)	5.9 (5.4)	4.0 (3.5)
s13	3.1 (2.6)	3.2 (2.6)	3.1 (2.6)	4.2 (3.3)	3.4 (3.3)	3.9 (2.9)	5.7 (5.0)	4.4(3.9)
s14	2.2 (1.8)	2.3 (1.9)	2.2 (1.8)	3.2 (2.3)	3.8 (2.8)	3.1 (2.5)	3.5 (3.0)	2.5 (2.1)
s15	2.3 (1.8)	2.3 (1.8)	2.3 (1.8)	4.2 (3.0)	3.8 (2.4)	3.5 (2.7)	3.3 (2.5)	3.8 (3.3)
Mean	3.0 (2.6)	3.4 (2.7)	3.0 (2.6)	4.1 (3.3)	4.1 (3.4)	3.7 (3.0)	5.3 (4.5)	5.1 (4.4)

*EIP* electric impedance plethysmography; *RIAV* respiratory induced amplitude variation; *RIFV* respiratory induced frequency variation; *RIIV* respiratory induced intensity variation; *RIMV* respiratory induced movement variation

* The best result

** The worst result

For all subjects, Fusion 1 and Fusion 3 have the lowest absolute mean error and similar performance. As for the five respiratory signals, the ranking in terms of absolute mean error are as follows: RIAV (3.7 rcpm), EIP (4.1 rcpm), RIFV (4.1 rcpm), RIMV (5.1 rcpm), and RIIV (5.3 rcpm). The movement artifacts deteriorate the quality of respiratory signal estimation using RIMV and RIFV, which though in static setting are more reliable than EIP, RIAV, and RIIV.

To compare the respiratory times of three fusion methods and CO_2_ concentrations, the Bland–Altman plots for three fusion algorithms in running task over 15 subjects are applied, as shown in Fig. 10d–f. The Bland–Altman plots show the difference between two different methods (In this case: the respiratory times from CO_2_ concentration and fusion method). The cycle-to-cycle interval (respiratory time) measured was used to compare with the reference. The X-axis is the mean of the respiratory times of CO_2_ concentration and fusion method and the Y-axis is the difference between CO_2_ concentration and fusion method. Comparing three fusion methods, Fusion 1 and Fusion 3 have better results than Fusion 2. The confidence limits for Fusion 2 is from −0.843 to 0.731 s, the confidence limits for Fusion 1 is from −0.76 to 0.713 s, and the confidence limits for Fusion 3 is from −0.762 to 0.709 s. Overall, the density of points from 1.5 to 3-s is similar except the outliers from 3 to 4-s. These are caused by the larger differences between the measured respiratory times and those of referred CO_2_ concentrations.

No matter in the sitting or the running experiment, Naïve Bayes inference (Fusion 1) and the dynamic Kalman filter (Fusion 3) have similar and better results than static Kalman filter (Fusion 2). Under the assumption of slow variation in respiratory signal, dynamic Kalman filter should have better results than Naïve Bayes inference, which is only considering the variance of respiratory signals. The current finding might be due to that the state space model we chose is too simple to represent the respiratory signal or that the coefficient of process noise covariance is not optimized. Because the RIIV has the worst result no matter in the static or running task, the extra investigation which fused only four respiratory signals without RIIV is done to see how the fusion methods perform. Those results are shown in the Table 5. No matter in the static or running task, the Fusion 2 without RIIV has the better result than Fusion 2. It is because the measurement noise covariance matrix is chosen as $R_{s}$ and it assumed the noise variances of each measurement signal are the same. After the experiments, it can be found the five measurement noise variances are totally different. Therefore, as the RIIV with largest variance was removed in the Fusion 2, the result is better. In the other hand, Fusion 1 and Fusion 3 updated the measurement noise variances in each time step by calculating the variances of the measuring respiratory rate in the window of past 30 s, therefore the current variances of RIIV are considered in Fusion 1 and Fusion3. The results show the mean of absolute error in Fusion 1 without RIIV is even a little worse than Fusion 1 in the static task. For a better result for static Kalman filter, the noise covariance matrix should be also tuned by offline data. We consider these potential improvements as our future work.

**Table 5:** The mean (standard deviation) of absolute respiratory rate error between three fusion methods with RIIV and without RIIV

Task	Fusion 1	Fusion 1 without RIIV	Fusion 2	Fusion 2 without RIIV	Fusion 3	Fusion 3 without RIIV
Static task	0.7 (1.0)	0.8 (1.4)	2.1 (1.5)	1.8 (1.6)	0.8 (1.0)	0.8 (1.4)
Running task	3.0 (2.6)	3.0 (2.6)	3.4 (2.7)	3.3 (2.6)	3.0 (2.6)	3.0 (2.6)

*RIIV* respiratory induced intensity variation

## Conclusions

In this study, multi-sensor wearable smart clothing is developed for measuring different human bio-sensors and sensor fusion is used to achieve better respiratory estimation. It can not only sense ECG, respiration, acceleration, and gyro, but also makes the respiratory rate estimation more accurate by data fusion. It is based on our novel textile electrodes, which have electric properties similar to the tradition electrodes and silver textile electrodes, but has reduced usage of silver fiber and therefore lower bio-toxicity and cost in manufacturing. In addition, we demonstrate that the proposed data fusion methods can combine multiple inferior respiratory sensors to reconstruct respiratory rate signals with lower error on average.

In the future, we plan to research in the adaptive peak detection for respiratory estimation because the accuracy of respiratory estimation highly depends on peak detection. Also, we will investigate in distributed sensor system. The system architecture in the current study is a central control system, in which a main controller receives data from sensors and sends command to a smart phone user interface, and therefore sensors need to be positioned together in the same controller box. For applications, such as continuous blood pressure measurement, EMG sensing, and human joint estimation, the central system will lead to very complicated connections: for example, the PPG sensor on the arm or forearm to calculate blood pressure; the EMG electrodes on each muscle belly; the accelerometer on each body segments to measure joint angles. Using a distributed sensor system allows sensors positioned anywhere on the body by integrating data directly to the cloud or smart phone.

Our current study provides a framework for sensor integration in smart clothing. Though the fusion methods in this study are unsupervised, supervised fusion methods based on probabilistic graphical model, Gaussian process, or support vector machine, can be developed for better respiratory rate estimation. Beyond respiratory rate estimation, this framework can also be applied to estimate ECG, heart rate, blood pressure, activity level, exercise performance, and sleep quality. We are interested in further developing these functions and their integration for daily life monitoring.

## Abbreviations

ECG: Electrocardiogram
EDR: ECG derived respiration
EIP: Electric impedance plethysmography
EMG: Electromyography
PAT: Peripheral arterial tonometry
PPG: Photoplethysmogram
RIAV: Respiratory induced amplitude variation
RIFV: Respiratory induced frequency variation
RIIV: Respiratory induced intensity variation
RIMV: Respiratory induced movement variation
RIP: Respiratory inductive plethysmography
TPM: Twists per meter
TPU: Thermoplastic polyurethane

## Footnotes

1 Carbon Black/polybutylene terephthalate/polyethylene yarn, CB/PBT/PET yarn, 70 denier 24 filaments, TTRI, Taiwan.

2 AGposs is with a core of Nylon and silver-plated yarn; AGposs, 74 denier 34 filaments, Mitsufuji Textile Ind. Co., Ltd, Japan.
